# Drug eluting and bare metal stents in people with and without diabetes: collaborative network meta-analysis

**DOI:** 10.1136/bmj.a1331

**Published:** 2008-08-29

**Authors:** Christoph Stettler, Sabin Allemann, Simon Wandel, Adnan Kastrati, Marie Claude Morice, Albert Schömig, Matthias E Pfisterer, Gregg W Stone, Martin B Leon, José Suárez de Lezo, Jean-Jacques Goy, Seung-Jung Park, Manel Sabaté, Maarten J Suttorp, Henning Kelbaek, Christian Spaulding, Maurizio Menichelli, Paul Vermeersch, Maurits T Dirksen, Pavel Cervinka, Marco De Carlo, Andrejs Erglis, Tania Chechi, Paolo Ortolani, Martin J Schalij, Peter Diem, Bernhard Meier, Stephan Windecker, Peter Jüni

**Affiliations:** 1 Institute of Social and Preventive Medicine, University of Bern, 3012 Bern, Switzerland; 2 Division of Endocrinology, Diabetes and Clinical Nutrition, University Hospital, Bern, Switzerland; 3 International Centre for Circulatory Health, National Heart and Lung Institute, Imperial College, London; 4 Deutsches Herzzentrum, Technische Universität, Munich, Germany; 5 Institut Hospitalier Jacques Cartier, Massy, France; 6 Division of Cardiology, University Hospital, Basel, Switzerland; 7 Columbia University Medical Center, New York, USA; 8 Servicio de Cardiología, Hospital Universitario Reina Sofía, Cordoba, Spain; 9 Service of Cardiology, Clinique Cecil, Lausanne, Switzerland; 10 Department of Medicine, ASAN Medical Center, Seoul, Korea; 11 Department of Interventional Cardiology, Hospital de San Pau, Barcelona, Spain; 12 Department of Interventional Cardiology, St Antonius Hospital, Nieuwegein, Netherlands; 13 Cardiac Catheterisation Laboratory, Rigshospitalet, Copenhagen, Denmark; 14 Cochin Hospital, Assistance Publique Hôpitaux de Paris, Paris Descartes University, and INSERM U 780, Paris, France; 15 San Camillo Hospital, Rome, Italy; 16 Antwerp Cardiovascular Institute Middelheim, Antwerp, Belgium; 17 Amsterdam Department of Interventional Cardiology, Onze Lieve Vrouwe Gasthuis, Amsterdam, Netherlands; 18 Division of Cardiology, Masaryk Hospital, Ústí nad Labem, Czech Republic; 19 Cardiothoracic and Vascular Department, University of Pisa, Italy; 20 University of Latvia, Institute of Cardiology, Riga, Latvia; 21 Cardiologia e Cardiologia Invasiva 2–AOU-Careggi, Florence, Italy; 22 Institute of Cardiology, S Orsola-Malpighi Hospital, University of Bologna, Italy; 23 Department of Cardiology, Leiden University Medical Center, Leiden, Netherlands; 24 Department of Cardiology, Bern University Hospital, Bern, Switzerland; 25 CTU Bern, Bern University Hospital, 3010 Bern, Switzerland

## Abstract

**Objective** To compare the effectiveness and safety of three types of stents (sirolimus eluting, paclitaxel eluting, and bare metal) in people with and without diabetes mellitus.

**Design** Collaborative network meta-analysis.

**Data sources** Electronic databases (Medline, Embase, the Cochrane Central Register of Controlled Trials), relevant websites, reference lists, conference abstracts, reviews, book chapters, and proceedings of advisory panels for the US Food and Drug Administration. Manufacturers and trialists provided additional data.

**Review methods** Network meta-analysis with a mixed treatment comparison method to combine direct within trial comparisons between stents with indirect evidence from other trials while maintaining randomisation. Overall mortality was the primary safety end point, target lesion revascularisation the effectiveness end point.

**Results** 35 trials in 3852 people with diabetes and 10 947 people without diabetes contributed to the analyses. Inconsistency of the network was substantial for overall mortality in people with diabetes and seemed to be related to the duration of dual antiplatelet therapy (P value for interaction 0.02). Restricting the analysis to trials with a duration of dual antiplatelet therapy of six months or more, inconsistency was reduced considerably and hazard ratios for overall mortality were near one for all comparisons in people with diabetes: sirolimus eluting stents compared with bare metal stents 0.88 (95% credibility interval 0.55 to 1.30), paclitaxel eluting stents compared with bare metal stents 0.91 (0.60 to 1.38), and sirolimus eluting stents compared with paclitaxel eluting stents 0.95 (0.63 to 1.43). In people without diabetes, hazard ratios were unaffected by the restriction. Both drug eluting stents were associated with a decrease in revascularisation rates compared with bare metal stents in people both with and without diabetes.

**Conclusion** In trials that specified a duration of dual antiplatelet therapy of six months or more after stent implantation, drug eluting stents seemed safe and effective in people both with and without diabetes.

## Introduction

People with diabetes experience a more generalised form of atherosclerosis than people without diabetes. They are at an increased risk for coronary heart disease and have more restenoses after the implantation of coronary stents. On average sirolimus eluting stents and paclitaxel eluting stents are associated with a noticeable reduction in target lesion revascularisation compared with bare metal stents, whereas the rates of overall mortality and cardiac mortality associated with the three stents are similar.^1^ Differences in the process and dynamics of restenosis along with variations in metabolic profiles may, however, alter safety or effectiveness profiles of the different stent types, particularly in people with diabetes.

Randomised trials have reported a reduced revascularisation rate with both sirolimus eluting stents and paclitaxel eluting stents compared with bare metal stents in people with diabetes,^2^ ^3^ ^4^^ w1-w3^ but the trials were hampered by small numbers of patients and a limited duration of follow-up. A meta-analysis of four early trials in 428 people with diabetes that compared sirolimus eluting stents with bare metal stents for up to four years suggested a strongly increased risk of mortality (hazard ratio 2.90, 95% confidence interval 1.38 to 6.10).^5^ In view of an average rate of overall mortality of 12% over four years observed in people with diabetes,^1^ this hazard ratio would translate into a number needed to harm to cause one death over four years as low as 4 (95% confidence interval 2 to 22). Another meta-analysis, published simultaneously,^6^ also included more recent trials with shorter durations of follow-up: pooling 14 trials in 1411 people with diabetes resulted in a hazard ratio of only 1.27, but the 95% confidence interval ranged from 0.83 to 1.95 and was compatible with both moderate benefit and substantial harm.

Network meta-analyses^7^ ^8^ or mixed treatment comparisons^9^ ^10^ ^11^ allow a unified, coherent analysis of all randomised controlled trials that compared either of the two drug eluting stents with bare metal stents or the two drug eluting stents head to head, while fully respecting randomisation. In a previous network meta-analysis^1^ we determined the average benefits and harms of all three stent types and provided preliminary results for overall mortality and the composite of death or myocardial infarction stratified according to the presence or absence of diabetes. Here we extend the network meta-analysis stratified according to diabetes status to include 35 trials in 14 799 patients, with data from five additional trials,^w4-w8^ a longer follow-up in one trial,^w7^ and data on cardiac death, myocardial infarction, stent thrombosis, and target lesion revascularisation as additional clinical outcomes.^12^ ^13^ To address earlier concerns^5^ we prespecified overall mortality as the primary safety outcome and systematically explored the consistency of mortality data in people with diabetes.

## Methods

We included randomised controlled trials in people with symptoms or signs of myocardial ischaemia as a result of coronary artery disease, that compared the two first generation drug eluting stents approved by the US Food and Drug Administration, a paclitaxel eluting stent (Taxus; Boston Scientific, Natick, MA) and a sirolimus eluting stent (Cypher; Cordis, Miami Lakes, FL) with each other or with a bare metal stent. Trials had to have a clinical follow-up duration of at least six months.

We searched Medline, Embase, the Cochrane Central Register of Controlled Trials (from inception of each database to October 2007), and relevant websites (www.acc.org, www.tctmd.com, www.theheart.org, www.clinicaltrialresults.org) for studies in any language. We checked reference lists, conference abstracts, relevant reviews, book chapters, and the proceedings of the relevant advisory panels of the Food and Drug Administration, and we contacted manufacturers and trialists. (See web extra appendix 1 for details of the search strategy.)

### Data extraction

Two investigators (CSt, SA) extracted data independently, with disagreements resolved in consultation with a third investigator (PJ). We asked the trialists and manufacturers of drug eluting stents to check the extracted information and to provide additional outcome data on an electronic form according to standardised definitions and attempted to obtain outcome data separately for people with and without diabetes.

We specified overall mortality as the primary safety outcome and target lesion revascularisation as the primary effectiveness outcome. Target lesion revascularisation was defined as repeat percutaneous intervention of the target lesion or bypass surgery of the target vessel done for restenosis or other complications of the target lesion (ranging from 5 mm proximal to 5 mm distal to the stent). We recorded the following secondary safety outcomes: cardiac death, defined as any death due to a cardiac cause (for example, myocardial infarction, low output failure, fatal arrhythmia), procedure related deaths, deaths related to concomitant treatment, and death of unknown cause; myocardial infarction, including fatal and non-fatal non-Q wave or Q wave myocardial infarction; a composite of death or myocardial infarction; and stent thrombosis, within the stented segment, confirmed by angiography or post-mortem examination in accordance with the criteria of the Academic Research Consortium, to ensure the inclusion of “secondary” stent thrombosis occurring after a patient had undergone a target lesion revascularisation. In addition, we recorded stent thrombosis according to the definitions used in individual trials (per protocol definition). The numbers of patients experiencing an event and the overall number of patients at risk were recorded separately for years 1 to 4.

To tackle potential sources of inconsistency in the network we determined the month of completion of patient recruitment as a measure of the period when a trial was done, the duration of follow-up in years, and the duration in months of dual antiplatelet therapy after stent implantation and assessed three key domains of internal validity^14^: concealment of allocation, blinding of research staff adjudicating clinical outcomes, and the inclusion of all randomised participants in the analysis according to the intention to treat principle. We considered trials to be of high quality that satisfied all three criteria (see also the criteria for quality assessment in the web extra, appendix 1). In most trials the precise durations of dual antiplatelet therapy in individual patients were unavailable. Therefore we obtained all relevant auxiliary information available for trials with a duration of dual antiplatelet therapy of less than six months, such as the information stated in the protocol and the percentage of patients reporting the use of dual antiplatelet therapy at relevant time points. On the basis of this information two investigators (CSt, PJ) independently classified trials according to the likely percentage of patients taking clopidogrel at six months, in increments of 10% (<10%, 10% to <20%, and so on).

### Statistical analysis

We used a hierarchical random effects model^15^ for mixed treatment comparisons^9^ based on piecewise constant hazards, with random effects at the levels of trials, adjacent time periods, and comparisons. We simultaneously estimated log hazard ratios for people with and without diabetes and the difference in log hazard ratios between such people. From the posterior distribution of the difference we estimated the P values for interaction between treatment effect and diabetes status. We excluded from the analyses time periods with zero events in both groups. Hazard ratios were estimated from the median, and the accompanying 95% credibility intervals from the 2.5th and 97.5th centiles of the posterior distribution. Because of low event rates we derived relative risks of stent thromboses using a previously described random effects Poisson regression model.^11^ As in the preceding study,^1^ we did separate analyses according to time of occurrence of stent thrombosis and an analysis of per protocol definitions of stent thrombosis as used in individual trials (see web extra appendix 1 for details of the models).

We evaluated the inconsistency of the network, defined as the variability of results across different comparisons of the network, by calculating inconsistency factors: the estimated difference between the log hazard ratios from direct comparisons within randomised trials and the log hazard ratios from indirect comparisons between randomised trials with one intervention in common.^1^ To allow intuitive interpretation we back transformed absolute values of these inconsistency factors to ratios of hazard ratios and expressed inconsistency as percentage difference in hazard ratios between direct randomised comparisons within trials and indirect comparisons between trials. Values can range from 0% to infinity. A value near 0 indicates that all the comparisons in the network are consistent, showing fully coherent estimates of hazard ratios comparing any two types of stent. The more the value deviates from 0% the more inconsistent the network. A value of 25%, corresponding to a ratio of hazard ratios of 1.25, may be interpreted to indicate low inconsistency, a value of 50% moderate inconsistency, and a value of 100%, corresponding to a ratio of hazard ratios of 2.00, high inconsistency (see web extra appendix 1). We evaluated heterogeneity between trials, defined as variability of results across trials within comparisons over and above chance, and the goodness of fit of the model to the data (see web extra appendix 1).

To investigate potential sources of variation in the network, we included the characteristics of the trials as covariates in the network meta-analysis of the primary safety outcome. We used prespecified cut-off points of two years for the length of follow-up, January 2004 for completion of patient recruitment, and six months for the duration of dual antiplatelet therapy. In some instances the numbers of trials and events were too low to allow the estimation of random effects at the level of time periods. Therefore we used a random effects Poisson regression model (see web extra appendix 1) for all of these analyses.^11^ P values for interaction between trial characteristics and treatment effect were derived from the posterior distribution of covariates. The duration of dual antiplatelet therapy specified in trial protocols was the only variable with a treatment by trial characteristic interaction at P<0.05. Therefore we restricted the dataset to trials with a duration of dual antiplatelet therapy of six months or longer and repeated all analyses. Heterogeneity between trials, defined as variability of results across trials within comparisons over and above chance, and the goodness of fit of the model to the data, were evaluated as previously reported (see web extra appendix 1).^1^ All analyses were done in WinBUGS version 1.4.1 and Stata version 9.2.

## Results

Forty two trials^w1-w42^ met the inclusion criteria (fig 1). Seven trials, totalling about 900 people with diabetes and 3000 without, were excluded because data stratified by diabetes status were not obtainable.^w15 w34 w36 w37 w39 w41 w42^ The remaining 35 trials^w1-w14 w16-w33 w35 w38 w40^ were included. Investigators or manufacturers provided additional data for 32 trials.^w1 w4-w14 w16-w19 w21-w33 w35 w38 w40^ (The characteristics of the 35 included trials are presented in web extra table A.) Four trials^w1-w3 w29^ included only people with diabetes and one trial only people without diabetes.^w33^ The trials had randomly allocated 3852 people with diabetes mellitus and 10 947 people without diabetes mellitus. Patient recruitment started between August 2000^w16^ and October 2004^w4^ and was completed between January 2001^w16^ and November 2005.^w5^ Data stratified according to the presence or absence of diabetes were available for all 35 trials on all outcomes, except stent thrombosis (see web extra tables B and C). Twenty nine trials described appropriate methods for allocation concealment,^w1 w2 w7 w8 w11-w14 w16-w33 w35 w38 w40^ and 28 trials reported blind adjudication for clinical outcomes.^w1 w4 w7 w9-w14 w16-w26 w28-w30 w32 w33 w35 w38 w40^ For 30 trials all randomised patients could be included in the analyses according to the intention to treat principle.^w1 w3-w7 w9 w11 w13 w14 w16-w23 w25-w33 w35 w38 w40^ Twenty four trials were considered to be of high quality.^w1 w7 w11 w13 w14 w16-w23 w25 w26 w28-w30 w32 w33 w35 w38 w40^

**Figure fig1:**
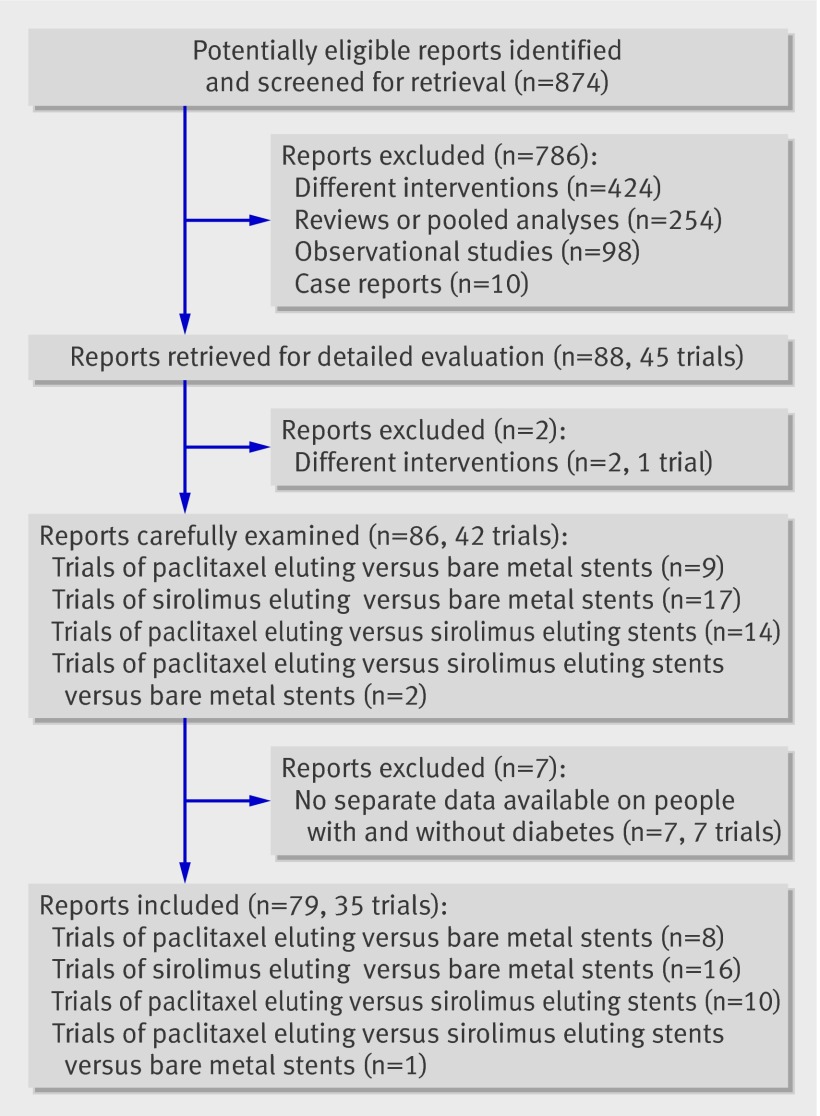
**Fig 1** Identification of trials

### Duration of dual antiplatelet therapy

The duration of dual antiplatelet therapy specified in the study protocols was two months in five trials,^w16 w18-w20 w26^ three months in three,^w3 w7 w17^ six months in 18,^w2 w5 w9-w14 w21 w22 w24 w28 w29 w32 w33 w35 w38 w40^ nine months in one,^w4^ and 12 months in eight.^w1 w6 w8 w23 w25 w27 w30 w31^ All eight trials with therapy lasting less than six months compared sirolimus eluting stents with bare metal stents. Auxiliary information for these trials (see web extra appendix 2) indicated that the percentage of patients actually receiving dual antiplatelet therapy of six months or more was likely to be below 10% in five trials.^w7 w16-w19^ The likely percentage for the other three trials was between 10% and 20%,^w20^ 40% and 50%,^w26^ and 50% and 60%.^w3^ One head to head comparison had specified a minimal duration of dual antiplatelet therapy of two months for sirolimus eluting stents and six months for paclitaxel eluting stents in the protocol, but the actual duration was only one month shorter for patients allocated to siroliumus stents than for patients allocated to paclitaxel stents, and about 50% of patients allocated to either stent type were still receiving therapy after eight months.^w32^

### Network of all trials: overall mortality

Table 1 presents the results of the network meta-analysis of overall mortality in people with and without diabetes. In people with diabetes the estimated hazard ratio for sirolimus eluting compared with bare metal stents was 1.14 (95% credibility interval 0.74 to 1.60), for paclitaxel eluting versus bare metal stents was 1.09 (0.71 to 1.66), and for sirolimus versus paclitaxel eluting stents was 1.02 (0.70 to 1.57), and compatible with both a substantial harm and a moderate benefit of either eluting stent compared with bare metal stents. The corresponding values for people without diabetes were 1.02 (0.77 to 1.29), 0.90 (0.67 to 1.16), and 1.13 (0.83 to 1.54; table 1). A moderate to high inconsistency of 61% was found among people with diabetes, but none among people without diabetes.

**Table 1 tbl1:** Analyses overall and restricted to trials with dual antiplatelet therapy of at least six months

Variable and stent types	People with diabetes						People without diabetes				
	All trials			Trials with dual antiplatelet therapy ≥6 months			All trials			Trials with dual antiplatelet therapy ≥6 months	
	Hazard ratio (95% CI)	IC*		Hazard ratio (95% CI)	IC*		Hazard ratio (95% CI)	IC*		Hazard ratio (95% CI)	IC*
Death overall:											
SES *v* bare metal stent	1.14 (0.74 to 1.60)	61%		0.88 (0.55 to 1.30)	20%		1.02 (0.77 to 1.29)	3%		1.05 (0.69 to 1.73)	1%
PES *v* bare metal stent	1.09 (0.71 to 1.66)			0.91 (0.60 to 1.38)			0.90 (0.67 to 1.16)			0.89 (0.66 to 1.18)	
SES *v* PES	1.02 (0.70 to 1.57)			0.95 (0.63 to 1.43)			1.13 (0.83 to 1.54)			1.23 (0.82 to 1.69)	
Cardiac death:											
SES *v* bare metal stent	1.09 (0.63 to 1.93)	24%		0.80 (0.42 to 1.57)	4%		0.88 (0.48 to 1.60)	10%		0.93 (0.45 to 1.81)	12%
PES *v* bare metal stent	1.08 (0.62 to 2.28)			0.94 (0.52 to 1.87)			0.90 (0.52 to 1.54)			0.89 (0.55 to 1.47)	
SES *v* PES	0.98 (0.46 to 2.08)			0.85 (0.50 to 1.55)			0.96 (0.58 to 1.84)			1.04 (0.54 to 1.95)	
Myocardial infarction:											
SES *v* bare metal stent	0.68 (0.44 to 1.05)	30%		0.68 (0.43 to 1.12)	30%		0.87 (0.64 to 1.20)	5%		0.81 (0.55 to 1.14)	20%
PES *v* bare metal stent	0.84 (0.55 to 1.31)			0.85 (0.54 to 1.43)			1.08 (0.74 to 1.51)			1.05 (0.72 to 1.42)	
SES *v* PES	0.79 (0.56 to 1.23)			0.80 (0.55 to 1.27)			0.81 (0.58 to 1.06)			0.75 (0.57 to 1.07)	
Death or myocardial infarction:											
SES *v* bare metal stent	1.04 (0.75 to 1.61)	57%		0.88 (0.57 to 1.27)	56%		0.93 (0.71 to 1.13)	6%		0.91 (0.69 to 1.13)	13%
PES *v* bare metal stent	1.07 (0.74 to 1.62)			0.91 (0.70 to 1.31)			1.04 (0.84 to 1.31)			1.00 (0.84 to 1.25)	
SES *v* PES	0.97 (0.72 to 1.34)			0.95 (0.69 to 1.27)			0.90 (0.71 to 1.09)			0.88 (0.71 to 1.06)	
Stent thrombosis (ARC definite):											
SES *v* bare metal stent	0.46 (0.15 to 1.42)	26%		0.33 (0.09 to 1.09)	24%		1.35 (0.76 to 2.73)	32%		1.24 (0.58 to 3.08)	20%
PES *v* bare metal stent	1.05 (0.32 to 4.01)			0.82 (0.23 to 3.09)			1.54 (0.83 to 3.13)			1.48 (0.69 to 3.40)	
SES *v* PES	0.44 (0.15 to 1.17)			0.40 (0.13 to 1.08)			0.87 (0.47 to 1.69)			0.84 (0.41 to 1.88)	
Stent thrombosis (per protocol):											
SES *v* bare metal stent	0.48 (0.17 to 1.35)	20%		0.20 (0.05 to 0.68)	5%		1.43 (0.78 to 3.00)	8%		1.48 (0.74 to 3.41)	10%
PES *v* bare metal stent	1.27 (0.38 to 4.91)			0.73 (0.19 to 2.80)			1.73 (0.88 to 3.61)			1.80 (0.89 to 3.67)	
SES *v* PES	0.38 (0.11 to 1.07)			0.27 (0.07 to 0.80)			0.82 (0.44 to 1.73)			0.82 (0.44 to 1.73)	
Target lesion revascularisation:											
SES *v* bare metal stent	0.29 (0.22 to 0.39)	2%		0.29 (0.19 to 0.45)	0%		0.29 (0.22 to 0.38)	3%		0.29 (0.19 to 0.42)	1%
PES *v* bare metal stent	0.38 (0.28 to 0.55)			0.38 (0.26 to 0.56)			0.46 (0.33 to 0.60)			0.46 (0.32 to 0.60)	
SES *v* PES	0.76 (0.53 to 1.05)			0.78 (0.50 to 1.14)			0.63 (0.49 to 0.82)			0.64 (0.49 to 0.84)	

SES=sirolimus eluting stent; PES=paclitaxel eluting stent; ARC=Academic Research Consortium.

*Inconsistency of network expressed as percentage difference in hazard ratios between direct randomised comparisons within trials and indirect comparisons between trials. Values near 0 indicate that all comparisons in network are consistent, showing fully coherent estimates of hazard ratios comparing any two stent types. Values can range from 0% to infinity. The more values deviate from 0%, the more inconsistent the network. A value of 25% may be interpreted to indicate low inconsistency, 50% moderate, and 100% high inconsistency. (Also see table 5 in web extra appendix 3 for 95% credibility intervals and P values of inconsistency estimates.)

### Exploration of sources of variation

Table 2 presents an investigation into potential sources of variation in people with diabetes in the network. Estimates of relative risk comparing sirolimus eluting stents with paclitaxel eluting stents depended to some extent on the quality of the trials, the length of follow-up, and the time of completion of patient recruitment (table 2), but 95% credibility intervals were wide and tests for interaction negative (P for interaction ≥0.16). The estimated relative risk of death when sirolimus eluting stents were compared with bare metal stents was greater when the specified duration of dual antiplatelet therapy was less than six months (2.37, 95% credibility interval 1.18 to 5.12) compared with six months or longer (0.89, 0.58 to 1.40, P for interaction 0.02), however. When three trials originally classified to have a short duration of dual antiplatelet therapy^w3 w20 w26^ were reclassified to have a duration of six months or longer, reflecting the auxiliary information indicating that more than 10% of patients in these trials were still receiving dual antiplatelet therapy at six months, differences were maintained.

**Table 2 tbl2:** Overall mortality in patients with diabetes: evaluation of variation in network according to different trial characteristics

Characteristic	SES *v* bare metal stent			PES *v* bare metal stent			SES *v* PES	
	Relative risk (95% CI)	P value for interaction		Relative risk (95% CI)	P value for interaction		Relative risk (95% CI)	P value for interaction
Concealment of allocation:								
Adequate	1.30 (0.86 to 2.02)	0.16		1.22 (0.74 to 1.99)	0.72		1.06 (0.69 to 1.67)	—
Unclear	0.32 (0.03 to 2.27)			0.93 (0.21 to 4.33)			—	
Blind adjudication:								
Yes	1.30 (0.84 to 2.16)	0.37		1.17 (0.67 to 1.96)	0.96		1.11 (0.69 to 2.04)	0.78
No	0.72 (0.17 to 2.46)			1.24 (0.10 to 11.76 )			0.94 (0.26 to 2.64)	
Intention to treat analysis:								
Yes	1.25 (0.81 to 2.02)	0.71		1.13 (0.65 to 1.92)	0.92		1.11 (0.71 to 1.87)	Not estimable*
No or unclear	0.97 (0.26 to 3.82)			1.08 (0.37 to 3.23)			0.14 (0.01 to 3.10)*	
High quality trial:								
Yes	1.40 (0.86 to 2.49)	0.27		1.28 (0.66 to 2.44)	0.61		1.08 (0.64 to 2.14)	0.80
No	0.70 (0.21 to 2.18)			0.97 (0.37 to 2.52)			0.93 (0.26 to 2.77)	
Length of follow-up:								
>2 years	1.37 (0.80 to 2.48)	0.51		1.30 (0.71 to 2.46)	0.54		1.05 (0.61 to 1.90)	0.97
≤2 years	1.01 (0.47 to 2.19)			0.91 (0.34 to 2.48)			1.02 (0.29 to 4.13)	
Patient recruitment:								
Completed Jan 2004 or later	0.93 (0.52 to 1.73)	0.93		1.11 (0.60 to 2.06)	0.54		0.83 (0.54 to 1.33)	0.94
Completed before Jan 2004	1.70 (0.96 to 3.08)			0.84 (0.41 to 1.81)			3.68 (0.41 to 32.9)*	
Dual antiplatelet therapy:								
≥6 months	0.89 (0.58 to 1.40)	0.02		0.97 (0.64 to 1.49)	—		0.92 (0.64 to 1.34)	—
<6 months	2.37 (1.18 to 5.12)			—			—	

A random effects Poisson regression model was used to derive relative risks from network; estimates may therefore differ slightly from those reported in table 1 and figure 2. *Relative risks for these comparisons are derived from one trial.

*Single trial had zero events in one arm.

### Restricted network: overall mortality

When the network was restricted to trials with dual antiplatelet therapy for six months or longer (table 1), the hazard ratios of death overall among people with diabetes were all below 1: sirolimus eluting stents compared with bare metal stents 0.88 (95% credibility interval 0.55 to 1.30), paclitaxel eluting stents compared with bare metal stents 0.91 (0.60 to 1.38), and sirolimus eluting compared with paclitaxel eluting stents 0.95 (0.63 to 1.43). Compared with the network of all trials the inconsistency decreased to 20% and credibility intervals of hazard ratios became more narrow in the restricted network. Among patients without diabetes results were much the same in the overall and the restricted network (table 1). The hazard ratio for sirolimus eluting stents compared with bare metal stents was 1.05 (0.69 to 1.73), for paclitaxel eluting stents compared with bare metal stents it was 0.89 (0.66 to 1.18), and for sirolimus compared with paclitaxel eluting stents it was 1.23 (0.82 to 1.69). Figure 2 [Fig fig2] presents corresponding cumulative incidences of death for the three stent types estimated from the restricted network meta-analysis separately for people with and without diabetes. The incidence of death was about twice as high in people with diabetes compared with people without diabetes. Tests for interaction between treatment effect and diabetes status were negative for all comparisons (P for interaction ≥0.28; also see web extra table D).

**Figure fig2:**
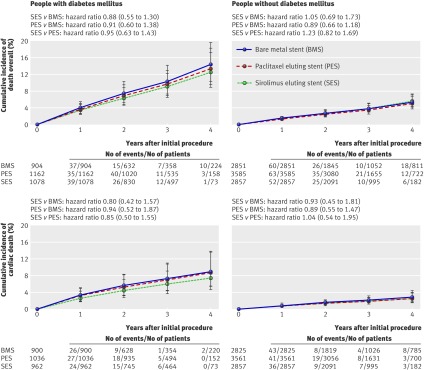
**Fig 2** Cumulative incidence of death overall and cardiac death and corresponding hazard ratios (95% credibility intervals) for three stent types estimated from network meta-analysis for pairwise comparisons in people with and without diabetes and restricted to trials with dual antiplatelet therapy of at least six months

### Restricted network: secondary safety outcomes

Table 1 allows a comparison of the results from the network meta-analysis of all trials and the analysis restricted to trials with a dual antiplatelet therapy of six months or more. Among people with diabetes, hazard ratios for drug eluting stents compared with bare metal stents became more beneficial for drug eluting stents for the outcomes of cardiac death, the composite of death or myocardial infarction, and for stent thromboses. The inconsistency decreased mainly for cardiac death and per protocol definitions of stent thromboses. No differences between overall and restricted network meta-analysis were observed for myocardial infarction. Among people without diabetes, results from overall and restricted network meta-analysis were similar. Corresponding cumulative incidences for the three stent types are presented in figures 2 and 3 [Fig fig3]. Again, incidences were higher in people with diabetes than without, with most pronounced differences observed for cardiac death. Tests for interaction between treatment effect and diabetes status were negative for all comparisons on cardiac death, myocardial infarction, and their composite (P for interaction ≥0.47, see also web extra table D).

**Figure fig3:**
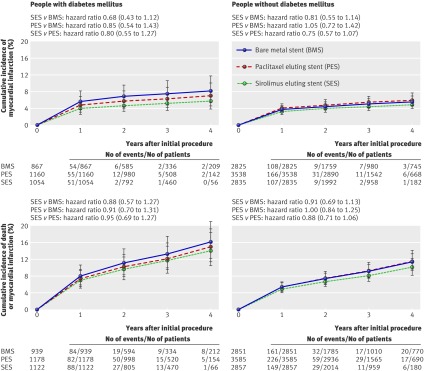
**Fig 3** Cumulative incidence of myocardial infarction and the composite of death or myocardial infarction and corresponding hazard ratios (95% credibility intervals) for three stent types estimated from network meta-analysis for pairwise comparisons in people with and without diabetes and restricted to trials with dual antiplatelet therapy of at least six months

Table 3 presents a breakdown of stent thromboses according to time of occurrence. Among people with diabetes little evidence was found for an increased risk of definite or per protocol stent thrombosis associated with sirolimus eluting stents compared with either of the two other stents; all point estimates were below 1 and differences in favour of sirolimus eluting stents became more pronounced with the use of per protocol definitions. For the comparison of paclitaxel eluting stents with bare metal stents all estimates were imprecise for both the Academic Research Consortium definition of definite stent thrombosis and per protocol definitions. Among people without diabetes relative risks were generally higher for both definitions, but tests for interaction between treatment effect and diabetes status were positive only for the comparison of sirolimus eluting stents with bare metal stents on per protocol definitions of stent thrombosis between day 0 and 4 years and between day 30 and 4 years (P for interaction=0.01, see also web extra table D).

**Table 3 tbl3:** Stent thromboses in trials with dual antiplatelet therapy of six months or more

Variable	Events					Relative risks (95% credibility interval)		
	BMS	PES	SES	Total		SES *v* BMS	PES *v* BMS	SES *v* PES
ARC definite stent thrombosis*								
Patients with diabetes:								
No of patients at risk	557	874	753	2184				
0 days to 4 years	13	17	9	39		0.33 (0.09 to 1.09)	0.82 (0.23 to 3.09)	0.40 (0.13 to 1.08)
0-30 days	11	9	6	26		0.25 (0.04 to 1.11)	0.39 (0.05 to 2.36)	0.60 (0.12 to 3.36)
>30 days to 4 years	2	8	3	13		0.72 (0.04 to 10.8)	3.54 (0.23 to 78.6)	0.20 (0.02 to 1.04)
Patients without diabetes:								
No of patients at risk	2439	3130	2647	8216				
0 days to 4 years	34	56	46	136		1.24 (0.58 to 3.08)	1.48 (0.69 to 3.40)	0.84 (0.41 to 1.88)
0-30 days	19	22	28	69		1.19 (0.43 to 3.09)	1.11 (0.38 to 2.97)	1.06 (0.41 to 2.90)
>30 days to 4 years	15	34	18	67		1.19 (0.43 to 4.13)	1.83 (0.67 to 5.85)	0.65 (0.26 to 1.70)
**Per protocol definition of stent thrombosis†**								
Patients with diabetes:								
No of patients at risk	723	912	870	2505				
0 days to 4 years	16	18	7	41		0.20 (0.05 to 0.68)	0.73 (0.19 to 2.80)	0.27 (0.07 to 0.80)
0-30 days	11	10	5	26		0.23 (0.03 to 1.08)	0.55 (0.09 to 3.05)	0.42 (0.07 to 1.89)
>30 days to 4 years	5	8	2	15		0.10 (0.01 to 0.93)	0.87 (0.06 to 10.3)	0.11 (0.01 to 0.75)
Patients without diabetes:								
No of patients at risk	2577	3382	2625	8584				
0 days to 4 years	29	58	46	133		1.48 (0.74 to 3.41)	1.80 (0.89 to 3.67)	0.82 (0.44 to 1.73)
0-30 days	22	24	28	74		1.11 (0.47 to 2.81)	0.99 (0.44 to 2.33)	1.15 (0.48 to 2.72)
>30 days to 4 years	7	34	18	59		2.29 (0.83 to 7.77)	4.12 (1.55 to 13.1)	0.55 (0.25 to 1.27)

BMS=bare metal stent; PES=paclitaxel eluting stent; SES=sirolimus eluting stent.

*According to Academic Research Consortium criteria.

†According to protocol definitions used in individual trials.

### Restricted network: target lesion revascularisation

Both drug eluting stents were robustly associated with a decrease in revascularisation rates compared with bare metal stents, network inconsistency was low, and results were unaffected by the restriction of the analysis to trials with a duration of dual antiplatelet therapy of six months or more in people with and without diabetes (table 1). Differences between sirolimus and paclitaxel eluting stents tended to be less pronounced among people with diabetes, and the credibility intervals overlapped 1 (fig 4). Tests of interaction between treatment effect and diabetes status were negative (P values for interaction ≥0.44, see also web extra table D).

**Figure fig4:**
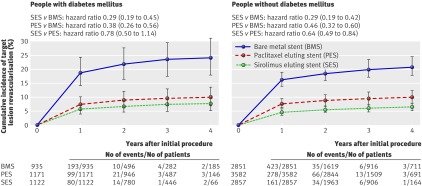
**Fig 4** Cumulative incidence of target lesion revascularisation and corresponding hazard ratios (95% credibility intervals) for three stent types estimated from network meta-analysis for pairwise comparisons in people with and without diabetes and restricted to trials with dual antiplatelet therapy of at least six months

### Between trial heterogeneity, model fit, and comparison with conventional meta-analyses

Web extra appendix 3 presents results from the evaluations of heterogeneity between trials and model fit. After restricting the network to trials with dual antiplatelet therapy of six months or more, heterogeneity between trials was low for all outcomes except stent thrombosis. For people without diabetes heterogeneity between trials was low for death, myocardial infarction, and the composite of death or myocardial infarction, and moderate for most comparisons on the other three outcomes. The model fit was adequate for all outcomes. Web extra table E presents a comparison of results from network meta-analyses with results from conventional meta-analyses: all estimates were compatible.

## Discussion

Our collaborative network meta-analysis suggests that previously reported increases in the risk of death associated with sirolimus eluting stents compared with bare metal stents in people with diabetes^5^ probably resulted from the restricted duration of dual antiplatelet therapy of less than six months in early trials. In trials with dual antiplatelet therapy for less than six months, the risk of death associated with sirolimus eluting stents was more than twice the risk associated with bare metal stents, which translates into a number needed to harm as low as 7 to cause one death over four years. Conversely, trials with dual antiplatelet therapy for six months or more showed no increase in risk from using sirolimus eluting stents compared with bare metal stents. Restricting the network to trials with dual antiplatelet therapy of six months or longer resulted in a clear reduction of the inconsistency and hazard ratios near 1, which were robust to all sensitivity analyses. We found similar patterns for analyses of cardiac death and stent thromboses. The restriction of the network resulted in a smaller evidence base: eight trials of 613 people with diabetes were excluded from the analysis. Despite this, statistical precision was improved owing to the accompanying decrease in the network’s inconsistency.

Compared with bare metal stents, target lesion revascularisation rates are strongly decreased by use of sirolimus and paclitaxel drug eluting stents in people with and without diabetes. Numbers needed to treat to reduce one event over four years are 6 in people with diabetes and 8 in people without diabetes. Active angiographic follow-up increases the absolute rates of target lesion revascularisation.^16^ ^17^ For example, the rate of target lesion revascularisation at one year in patients who had undergone implantation of a bare metal stent in the TAXUS IV trial was 18.9% in those allocated to active follow-up and 14.3% in those allocated to regular clinical follow-up without mandatory angiography. The number needed to treat to avoid one revascularisation would therefore be somewhat lower in clinical routine. Assuming revascularisation rates of 12% in people with diabetes and 9% in people without diabetes, as found in the Cardiac Care Network of Ontario at two years,^18^ numbers needed to treat were estimated as 13 for people with diabetes and 18 for people without diabetes.

Our study comprises a large body of evidence from randomised controlled trials in people with and without diabetes treated with one of two drug eluting stents or bare metal stents. Investigators and manufacturers provided additional data according to uniform outcome definitions, including a standardised definition for stent thrombosis according to the Academic Research Consortium consensus.^13^ This increases comparability between trials and limits bias, such as the censoring of events after intervening revascularisation.

Our previous network meta-analysis^1^ was recently criticised for integrating evidence from direct and indirect comparisons, for not comparing like with like, and for using random effects models that give undue weight to small studies.^19^ Our model was based on relative treatment effects (log hazards ratio), and variations in characteristics of patients or lesions between trials are fully accounted for in the analysis by maintaining randomised comparisons within each trial.^1^ Network meta-analysis makes similar assumptions to standard meta-analysis of direct comparisons within trials, but requires that these assumptions hold over the entire set of trials in the network.^10^ The smaller the heterogeneity between trials and the smaller the inconsistency of the data, the more likely relative treatment effects originate from the same distribution and less likely small trials get undue weight in the analysis. Therefore a careful exploration of heterogeneity between trials and inconsistency of the data is mandatory. Inspecting the entire network allowed us to gain insights into potential sources of variation, which could not have been achieved by an isolated look at just one set of trials comparing only two stent types. This led us to suggest a plausible explanation for the previously reported increase in the risk of death associated with sirolimus eluting stents compared with bare metal stents in people with diabetes^5^—that is, a restricted duration of dual antiplatelet therapy particularly in early trials.

Our exploration of inconsistency is observational in nature and has the same limitations as other observational studies.^20^ Most importantly, earlier trials had specified shorter durations of dual antiplatelet therapy than later trials. The duration of therapy was therefore bound to be negatively correlated with the duration of follow-up, and confounding could exist between the duration of therapy and the length of follow-up. Other potential confounders include changes over time in patient selection and procedural characteristics, such as an under-sizing or under-expansion of stents in early trials, or methodological quality. We addressed this by repeating tests of interaction between treatment effect and components of methodological quality or length of follow-up after the exclusion of trials with a duration of dual antiplatelet therapy of less than six months and found no evidence for an interaction in any of these analyses (data available on request).

We acknowledge that our results could be corroborated by an analysis of the actual duration of dual antiplatelet therapy in individual patients, but precise durations in individual patients are unavailable in most trials and we lacked the resources to retrospectively ascertain and validate usage data. Eight trials had specified a duration of dual antiplatelet therapy of less than six months. For five trials we are confident that the actual duration of dual antiplatelet therapy corresponded to the specified duration in at least 90% of patients^w7 w16-w19^; in three trials, however, between 10% and 50% of the patients had dual antiplatelet therapy at six months.^w3 w20 w26^ Results were unaffected by the reclassification of these three trials to have a duration of dual antiplatelet therapy of six months or longer, and the P value for interaction between relative risk of death and duration of dual antiplatelet therapy became even smaller. Additionally, strut thickness or type of bare metal stent used in comparison groups might affect clinical outcomes.^w21^ Even though our results are robust to the adjustment for these characteristics of bare metal stents,^1^ we cannot fully exclude the possibility that differences in bare metal stents as comparators contributed to the observed variation in mortality between trials with short and long durations of dual antiplatelet therapy. Four trials included only people with diabetes^w1-w3 w29^ and one trial only people without diabetes.^w33^ Performing network meta-analyses separately for people with and without diabetes allowed us to also incorporate these trials in our analysis. An alternative approach would have been to model differences between people with and without diabetes directly within each trial, but at the price of excluding these five trials.^w1-w3 w29 w33^ A final limitation of our study is that we were unable to record information on specific antidiabetic treatment or on glycaemic control in people with diabetes mellitus and to perform separate analyses for people with diabetes who did or did not use insulin. Although these aspects are related to cardiovascular outcomes,^21^ they were per definition randomly distributed across comparison groups within each trial and it seems unlikely that they influenced results.

We found that the duration of dual antiplatelet therapy modified the safety profile of drug eluting stents mainly in people with diabetes. The beneficial effect of prolonged therapy in people with diabetes may be mainly related to differences in lesion characteristics. People with diabetes tend to have smaller vessels and longer lesions than people without diabetes and therefore require stents of smaller size and longer length. Previous studies have identified both vessel size and lesion length as predictors of stent thrombosis,^22^ which may explain the predisposition of people with diabetes to this adverse event in the absence of adequate antiplatelet therapy. In necropsy studies, drug eluting stents delayed arterial healing and re-endothelialisation compared with bare metal stents.^23^ ^24^ Again, this effect may be enhanced in people with diabetes, particularly in the absence of dual antiplatelet therapy. Moreover, overall longer stents in people with diabetes may result in a prolongation of the healing and re-endothelialisation process. Finally, people with diabetes may be more likely to experience aspirin resistance than people without diabetes^25^ and may have a particular benefit from a complementary antiplatelet therapy with clopidogrel or ticlopidine. Taken together, these mechanisms may explain why prolonged dual antiplatelet therapy may be particularly important in people with diabetes.

We recently reported a potentially reduced average risk of myocardial infarction associated with sirolimus eluting stents compared with bare metal stents but not with paclitaxel eluting stents compared with bare metal stents.^1^ Our analysis suggests that this effect is likely to be present in people with diabetes, but wide credibility intervals of stratified analyses preclude firm conclusions. Stone et al found that about 1.4% of patients undergoing target lesion revascularisation developed a myocardial infarction associated with the procedure.^26^ A stent that decreases revascularisation rates will therefore result in a reduction of procedure related myocardial infarctions but this will result in a maximum reduction of the relative risk of about 4%, whereas the actually observed average relative risk reduction was 19%.^1^ Other mechanisms will therefore have to be considered. The process of restenosis in itself may result in myocardial infarction in some patients,^27^ and a more pronounced reduction of restenosis of sirolimus eluting stents may directly contribute to decreasing the risk of myocardial infarction. Although stent thrombosis is uncommon it is associated with a high incidence of myocardial infarction,^26^ and the benefits of drug eluting stents for myocardial infarctions are less likely to be offset by stent thromboses in sirolumus eluting stents than in paxlitaxel eluting stents.^1^

The mechanisms of action of the two drugs merit some consideration. Sirolimus, a macrocyclic lactone, inhibits mammalian target of rapamycin (mTOR) thereby blocking cell division by interfering at the transition from G1 to S phase.^28^ People with diabetes show a breakdown in the phosphatidylinositol 3 kinase insulin signal transduction pathway, where mTOR has an important role,^29^ and this has been thought to limit the effectiveness of sirolimus in people with diabetes mellitus. Paclitaxel, which affects cell replication by stabilising microtubules, does not seem to be influenced by insulin resistance and was therefore hypothesised to be superior to sirolimus in people with diabetes. The present study does not support these speculations: although paclitaxel eluting stents tended to reduce the risk of target lesion revascularisation more strongly in people with diabetes than in people without diabetes, sirolimus eluting stents were superior to paclitaxel eluting stents in reducing the risk of revascularisation in people both with and without diabetes.

Rapid technological developments led to novel, second generation drug eluting stents using different antiproliferative substances, absorbable polymers used as drug carriers, and fully bioabsorbable stent systems. Although the short term results for effectiveness of these new generation devices are encouraging,^30^ ^31^ ^32^ their long term safety profile in people with and without diabetes is still ill defined. An expansion of the present network meta-analysis will be required as soon as longer term follow-up data become available for these novel stent systems.

### Conclusion

In trials with a duration of dual antiplatelet therapy of six months or longer drug eluting stents were safe and effective in people with and without diabetes. It seems prudent to adhere to a minimal duration of dual antiplatelet therapy of six months in patients undergoing implantation of a drug eluting coronary stent. The potential benefits of a longer duration of therapy need to be balanced against potential risks, such as clinically relevant bleeding. The optimal duration can only be determined in adequately powered large scale randomised controlled trials.

**What is already known on this topic**

People with diabetes are at an increased risk for coronary heart disease and have more restenoses after the implantation of coronary stents
A meta-analysis suggested a strongly increased risk of death associated with sirolimus eluting stents compared with bare metal stents in people with diabetes

**What this study adds**

Reported increases in the risk of death associated with drug eluting stents compared with bare metal stents in people with diabetes were probably due to dual antiplatelet therapy lasting less than six months in early trials
In trials with dual antiplatelet therapy for six months or longer drug eluting stents were safe and effective in people both with and without diabetes
In clinical practice it seems prudent to adhere to a minimal duration of dual antiplatelet therapy of six months in all patients undergoing implantation of a drug eluting coronary stent

## Notes

We thank Boston Scientific and Cordis for the provision of additional data. CTU Bern is supported by the Swiss National Science Foundation.

Contributors: CSt and SA contributed equally to the manuscript. PJ and CS conceived the study. PJ, CSt, SWa, SA, and SWi were responsible for the conception and design of the study. SWa, CSt, SA, and PJ did the analysis and interpreted the analysis in collaboration with SWi. CSt, SA, AK, MCM, AS, MEP, GWS, MBL, JSdL, JJG, SJP, MS, MJS, HK, CSp, MM, PV, MTD, PC, ASP, AJN, PD, BM, SWi, and PJ were responsible for the acquisition of data. PJ, CSt, SA, and SWi wrote the first draft of the manuscript. All authors critically revised the manuscript for important intellectual content and approved the final version of the manuscript. CSt and PJ obtained public funding. PJ, CSt, PD, BM, and SWi provided administrative, technical, and logistic support.

Ethical approval: Not required.

Provenance and peer review: Not commissioned; externally peer reviewed.

## Notes

**Cite this as:** *BMJ* 2008;337:a1331
